# A seat at the table is not enough: a perspective on Black women representation in academia

**DOI:** 10.1111/imcb.12584

**Published:** 2022-09-25

**Authors:** Ane Ogbe

**Affiliations:** ^1^ Peter Medawar Building for Pathogen Research University of Oxford Oxford UK

## Abstract

This article is a personal perspective on the double‐dose effect of racism and sexism on Black females within the academic system. I present statistical evidence of the under‐representation of this group in academic leadership positions and discuss some factors – systematic and cultural – that have contributed to the low numbers of Black women in academic leadership. The detrimental impact of this under‐representation is supported by anecdotes from other Black women in academia. Finally, I propose some practical solutions to increase the representation of Black women in academia through the proactive inclusion of Black women in the design of frameworks and policies targeted to improve racial and gender‐based inequality.

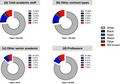

When I was asked to write a commentary on my experience in academia, I knew the experience of Black females in academia was one I wanted to write about. Still, I agonized over whether I was the best person to write this. I wondered whether I had the right to speak on behalf of many Black women in academia, and whether my lived experience was sufficient. I resolved that it was an important issue that needs to be highlighted. As such, I would add my voice to those of many others. I had been given a platform to sound it from, and that was enough (Figure 1).

**Figure 1:**
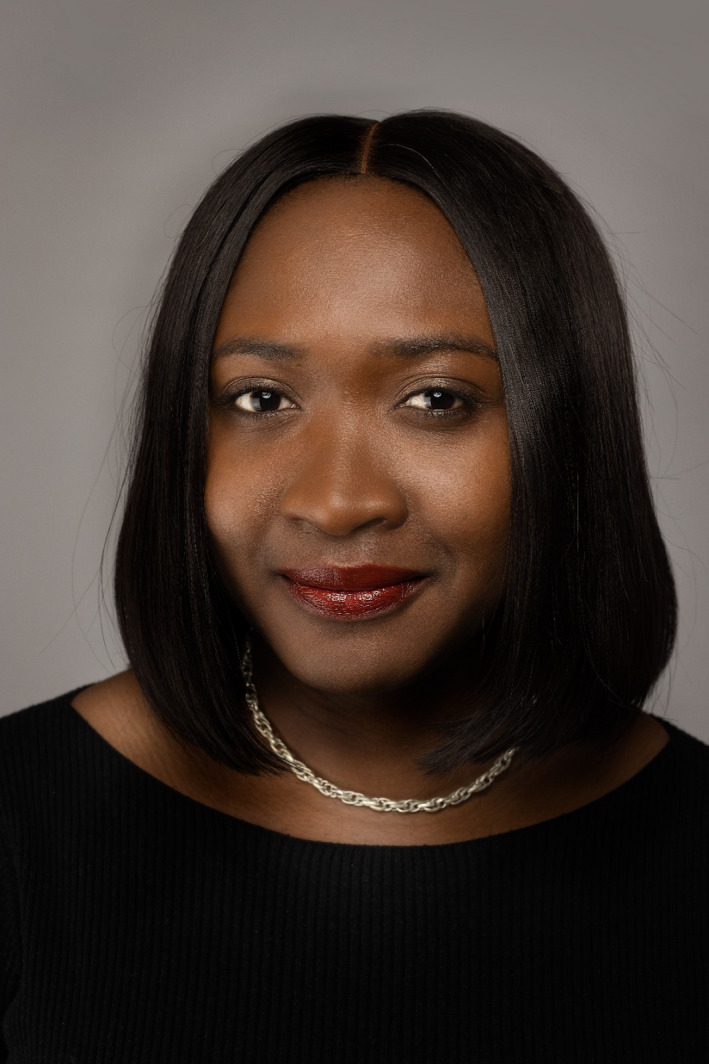
Dr Ane Ogbe. [Colour figure can be viewed at wileyonlinelibrary.com]

Although it has been reported extensively that academia has a racism^1^, ^2^, ^3^, ^4^ and sexism^5^, ^6^, ^7^ problem, the intersection of race and gender in academia is one that is not often highlighted and thus unacknowledged. The reality is that racism and sexism have a double‐dose effect for Black women in academia. Studies on these social ills have focused broadly on race and gender;^7^ therefore, the intervening measures put in place to tackle racism and sexism disproportionately favor Black men and White women, respectively, leaving Black women altogether forgotten.^8^ This is true for academia as it is with other industries where archaic and elitist structures persist. Within these systems, measures that facilitate inclusion and diversity are not naturally woven into the structural and cultural frameworks of the establishment. Published annual reports from various institutions present the veneer of improved racial and gender diversity in academia,^9^, ^10^, ^11^ but there is still work to be done on racism and sexism in these institutions. Part of that work is to give our focus on these social inequalities some more depth.

The statistics for Black women's involvement in academia are as depressing as they are alarming. According to the 2011 England and Wales census, 3.3% of the population identify as Black, with females making up at least half of this population.^12^, ^13^ The Higher Education Statistic Agency (HESA) reported that in 2021 there were over 220 000 academics (at all levels) in the United Kingdom; however, only 2% of them were Black.^14^ At higher academic levels there were over 22 000 professors in the United Kingdom, with only 160 (0.7%) Black professors^14^ (Figure 2). Of these Black professors, there were only 35 Black female professors.^15^ Black female professors represented 0.15% of UK professors in 2021. Clearly, Black women in leadership positions in academic research are very much under‐represented. I often of what factors are responsible for our recruitment into, retention and progression in academic careers. One factor that I believe has immense impact and far‐reaching consequences is the representation (or lack of representation) of Black women in academia.

**Figure 2:**
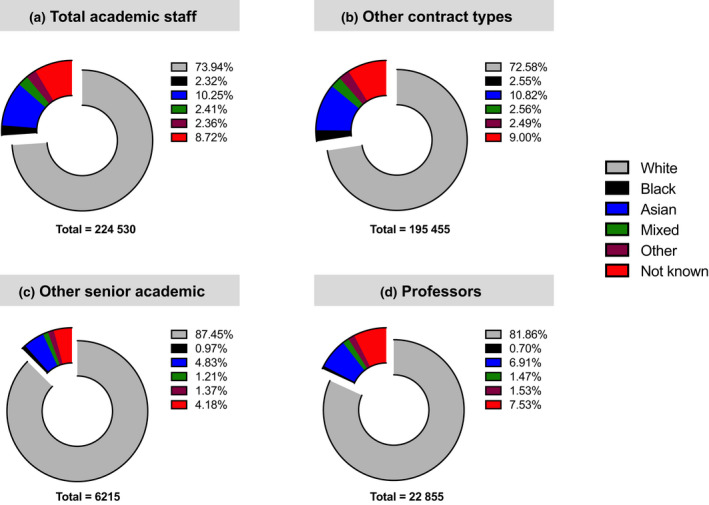
Black people are under‐represented at the top of academic leadership. **(a–d)** 2021 data from the Higher Education Statistic Agency (HESA) showing frequency of staff (male and female) who identify as members of particular ethnic groups in academia. Total academic staff is the sum of all people employed in typical academic roles including managers, directors, professors, associate professionals, clerks and technical and manual operators. Other contract types include staff at professional occupations which do not have the title of professor or have departmental line management responsibilities. Other senior academics are senior members of staff with departmental line management responsibilities but do not carry the title of professors. As a result of method of data collection, there may be some redundancy between professors and senior academics. [Colour figure can be viewed at wileyonlinelibrary.com]

The Latin word *repraesentare* from which representation is derived makes it clear. It is to make present; to have in sight. Hanna Fenichel Pitkin described its evolution to the modern meaning of the word as being interwoven with modern (English and American) history. Representation was deemed the “right of man … of every man's right to have a say in what happens to him”.^16^ The Oxford Learner's dictionaries define it as “the fact of having someone who would speak or vote for you”.^17^ These definitions encompass representation as I see it: a right to an advocate. In academia, Black women are a minority and often lack the organizational powers of the dominant White and male cultures. It is therefore important that someone speaks for the Black woman and advocates for her inclusion.

There are several reasons, anecdotal and empirical, why representation matters particularly in the academic system where career progression is not defined or necessarily meritocratic and bullying is rife.^18^, ^19^ Informal social networks and social capital acquired through representation play a role in providing the opportunities that would facilitate career success.^20^, ^21^ This complementary pathway to career success is lacking for Black women in academic environments. Furthermore, representation provides psychological support and a feeling of belonging achieved through the commonalities shared by people with similar backgrounds, cultural characteristics and experiences such as their shared impediments to progression in the workplace.^22^ Equitable access to these social networks, psychological support and mentorship obtained through networks of other Black women, are necessary for us to feel supported in an academic environment.

Narratives of the lived experience of Black women in academia abound. Social media platforms such as Twitter have become popular among academics. This has provided an opportunity for all academics to share, in addition to their research, the woes that surround our experience as academics. These conversations on social media platforms have highlighted the negative experience of many black women in academia. What was previously thought to be isolated cases of Black women not feeling supported in their academic career development, and feeling pressured to do more work in order to get similar recognition afforded their White peers or men for significantly less work, has become a collective cry. It is universal for us, and it is a problem. Personal conversations with Black female colleagues in different institutions have confirmed this. One colleague spoke of feeling invisible. Her good works, she said, were never credited to her without her “making a fuss”. This fuss, however, gets noticed very quickly and then she becomes “that angry Black woman”. She says her fear of reinforcing the angry Black woman stereotype has kept her from taking credit for some of her work. Unfortunately, many Black women feel this way too often. The question many of us ask is: why do we have to be put in a position to constantly be on the defensive? As Black women, we are more likely to experience bullying, harassment,^23^ feel unsupported and navigate a work environment replete with acts of microaggression and racism.^24^ Despite this, we are more likely to be labeled the aggressor.^25^ It would appear in some cases that our intellectual ability is judged from the way we speak, wear our hair and our physical appearance.^25^ To many of us, our physical appearance has now become a tool used in our oppression, causing us to code‐switch and present an unauthentic self at work.^25^, ^26^, ^27^ It is under these conditions that Black female academics have to work and try, if at all possible, to thrive without an advocate for our cause. It is easy to see why one would choose self‐preservation over career progression in these circumstances.

There are volumes to be written about the hurdles faced by Black women in academia but how do we move forward from where we are? It will require no small effort if academia is to improve the experience and progression of Black women academics. Academic institutions would need to proactively increase the recruitment, retention and progression of this group within its system as a major focus. Black women academics must be involved in the policy and decision‐making process within their respective academic institutions, not in perfunctory roles, but as genuine stakeholders at every level of the academic discourse. One of the benefits of such stakeholder engagement in policy making and its implementation is the inclusion of members of a community in advocating for their own futures.^28^ Involving Black women as active stakeholders in deciding how to support their academic careers within the academic system would promote transparency and trust in the process.^28^, ^29^ Indeed, such inclusion, akin to those of community representatives in human research study designs and clinical trials, could lead to better retention of Black women in academia. Correspondingly, policies and frameworks that systematically discriminate against this group should be reviewed and repealed. This is exemplified by the evolution of the UK Race Relation Acts from its initiation in 1965 to an amendment in 2000 and finally, the consolidated Equality Act of 2010.^30^ Finally, the aggregation of data on Black, Asian and minority ethnic (BAME) groups reduces granularity in the interpretation of the impact of social, economic, behavioral and cultural factors on specific populations.^31^, ^32^ To mitigate this, data specifically focused on the experience of Black women in academia should be collected and interpreted within context, to allow for the designing of effective policies to benefit and protect this group.

To conclude, a pathway to foster sustainable representation for Black women in academic spaces, through a culture of inclusivity and safety, must be forged. Indeed, a seat at the table is not enough if it is just tokenism.

## CONFLICT OF INTEREST

The author declares no conflict of interest.

## Data Availability

Data sharing is not applicable to this article as no new data were created or analyzed in this study.
